# Reproducible and Interpretable Machine Learning-Based Radiomic Analysis for Overall Survival Prediction in Glioblastoma Multiforme

**DOI:** 10.3390/cancers16193351

**Published:** 2024-09-30

**Authors:** Abdulkerim Duman, Xianfang Sun, Solly Thomas, James R. Powell, Emiliano Spezi

**Affiliations:** 1 School of Engineering, Cardiff University, Cardiff CF24 3AA, UK; espezi@cardiff.ac.uk; 2 School of Computer Science and Informatics, Cardiff University, Cardiff CF24 4AG, UK; sunx2@cardiff.ac.uk; 3 Maidstone and Tunbridge Wells NHS Trust, Kent ME16 9QQ, UK; solly.thomas@nhs.net; 4 Department of Oncology, Velindre University NHS Trust, Cardiff CF14 2TL, UK; james.powell2@wales.nhs.uk

**Keywords:** magnetic resonance imaging (MRI), radiomics, machine learning, clinical applications, glioblastoma multiforme

## Abstract

**Simple Summary:**

This study aimed to develop and validate a radiomic model for predicting overall survival (OS) in glioblastoma multiforme (GBM) patients using pre-treatment MRI images. A retrospective dataset of 289 patients from multiple institutions was used to extract 660 radiomic features (RFs) from each patient’s tumor volume. The initial model was enhanced by incorporating clinical variables and validated through repeated three-fold cross-validation. The final clinical–radiomic model utilized primary gross tumor volume (GTV) and T2-FLAIR MRI modality and includes the age variable and two robust RFs. The model achieved a moderately good discriminatory performance (C-Index: 0.69) and significant patient stratification (*p* = 7 × 10^−5^) on the validation cohort. Notably, the trained model exhibited the highest integrated area under curve (iAUC) at 11 months (0.81) in the literature. The study concluded that the validated clinical–radiomic model can effectively stratify GBM patients into low and high-risk groups based on OS. Future work will focus on integrating deep learning-based features and standardized convolutional filters to improve OS predictions.

**Abstract:**

Purpose: To develop and validate an MRI-based radiomic model for predicting overall survival (OS) in patients diagnosed with glioblastoma multiforme (GBM), utilizing a retrospective dataset from multiple institutions. Materials and Methods: Pre-treatment MRI images of 289 GBM patients were collected. From each patient’s tumor volume, 660 radiomic features (RFs) were extracted and subjected to robustness analysis. The initial prognostic model with minimum RFs was subsequently enhanced by including clinical variables. The final clinical–radiomic model was derived through repeated three-fold cross-validation on the training dataset. Performance evaluation included assessment of concordance index (C-Index), integrated area under curve (iAUC) alongside patient stratification into low and high-risk groups for overall survival (OS). Results: The final prognostic model, which has the highest level of interpretability, utilized primary gross tumor volume (GTV) and one MRI modality (T2-FLAIR) as a predictor and integrated the age variable with two independent, robust RFs, achieving moderately good discriminatory performance (C-Index [95% confidence interval]: 0.69 [0.62–0.75]) with significant patient stratification (*p* = 7 × 10^−5^) on the validation cohort. Furthermore, the trained model exhibited the highest iAUC at 11 months (0.81) in the literature. Conclusion: We identified and validated a clinical–radiomic model for stratification of patients into low and high-risk groups based on OS in patients with GBM using a multicenter retrospective dataset. Future work will focus on the use of deep learning-based features, with recently standardized convolutional filters on OS tasks.

## 1. Introduction

Glioblastoma multiforme (GBM) is a fatal primary brain neoplasm [1], classified as a Grade IV glioma due to its aggressive nature and propensity for rapid progression. The median survival for patients with GBM is approximately 15 months after the initial diagnosis [2]. The poor prognosis may be attributed to the considerable genetic heterogeneity observed within GBM tumor tissue [3]. Accurate prediction of a patient’s overall survival (OS) is important for guiding the optimal selection of treatment and management strategies. In current practice, a range of factors are used to predict the prognosis of patients with GBM; these include patient factors such as age and performance status and tumor factors such as the molecular genetic tumor profile, dimensions of the neoplasm prior to surgery and ability to maximally resect the tumor [4]. However, the subjective assessment of some of these factors highlights the increasing necessity for objective and quantitative radiological assessment.

Neurosurgical procedures, important for diagnosis and characterizing brain tumors [5], can be limited by tumor location, their invasiveness and sampling scope and can potentially miss genetic diversity within tumors [6]. Non-invasive quantitative imaging analysis could complement biopsies by evaluating the entire tumor and may substitute them when they are unsafe or not viable [7]. Magnetic resonance imaging (MRI) plays a pivotal role in neuro-oncology for both diagnoses and the assessment of treatment response, offering detailed tumor visualization without ionizing radiation and providing superior soft tissue contrast compared to X-ray and computer tomography (CT) scans [8]. Radiomic analysis is an expanding field in medical imaging as it deals with the extraction of quantitative features and texture variations of the radiological images to uncover patterns not visible to the naked eye. It utilizes advanced imaging techniques to assess tumor heterogeneity [9] and the microenvironment [10]. The radiomic features are to train models using statistical and machine learning (ML) methods for classification and regression tasks, as detailed in the literature [11]. Radiomics can play an important future role in enhancing personalized and precise patient care [12,13].

For time-to-event survival analysis of GBM, prior research demonstrated the effectiveness of radiomics based on MRI for risk stratification [14,15,16,17]. Previous radiomic models often incorporated a large number of features, which presented challenges in interpretation and risk for overfitting, deviating from established radiomic guidelines [18]. There is a need for a radiomic-based model for GBM that incorporates a minimum set of stable and interpretable features, similar to those developed for other cancer types [19,20].

In this study, we seek to bridge this gap by prioritizing reproducible, stable features and interpretable ML models with a minimal number of radiomic features (RFs) to stratify GBM patients into high and low-risk groups based on survival information. We consider limitations in dealing with real-world data such as reliance on a single region of interest (ROI), the gross tumor volume (GTV) used in neuro-oncology radiotherapy, access to a limited number of MRI sequences and variable MRI acquisition parameters across patients.

## 2. Materials and Method

### 2.1. Study Population

In this study, radiomic signatures were developed and validated based on 289 patients with GBM. The study included two datasets: (1) the publicly available BraTS (Brain Tumor Segmentation) Challenge 2020 including 236 GBM cases [21,22,23] and (2) a local dataset called STORM_GLIO, a retrospective collection of patients with a diagnosis of GBM treated at our institution between April 2014 and April 2018. The STORM_GLIO dataset included 53 eligible cases from a total of 108 patients. The four preoperative MRI sequences included in both datasets were T1-weighted (T1), T1-weighted contrast-enhanced (T1ce), T2 weighted (T2) and T2 fluid attenuated inversion recovery (T2-FLAIR) following the guidelines of the response assessment in the neuro-oncology (RANO) working group [24]. Both datasets also provided OS and age information.

### 2.2. Study Design

**Figure 1:**
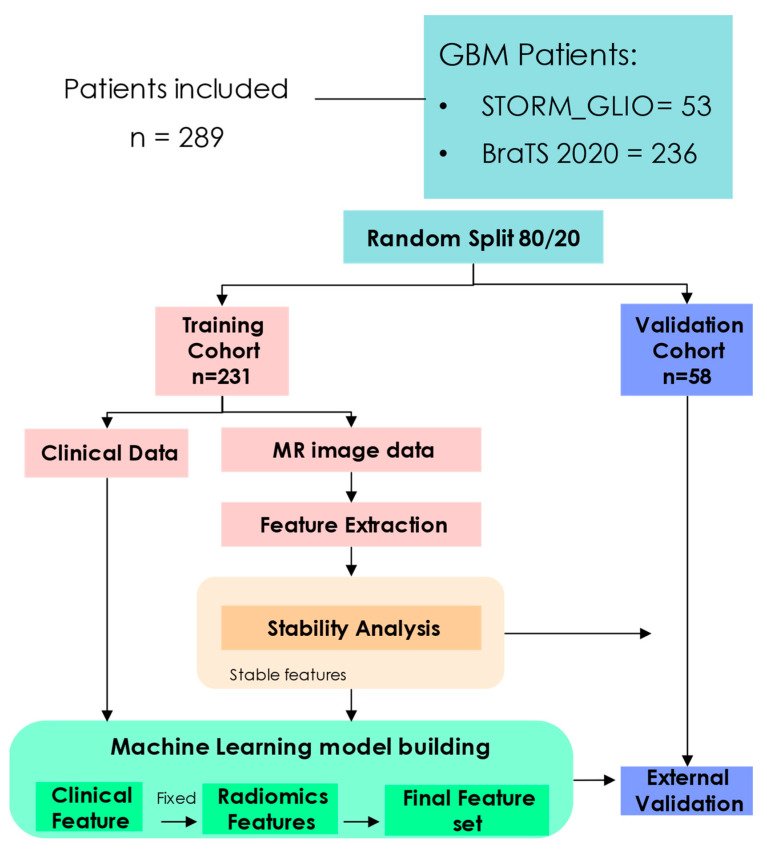
The workflow of the study: dataset splitting, feature extraction, stability analysis, model building, model validation.

Figure 1 shows the study design. A time-to-event task, assessing OS, was defined as the duration in days from the initial pathological diagnosis to either the date of death (censored = 1) or the last known date that the patient was alive (censored = 0). Patients were randomly allocated into training and validation datasets using an 80/20 ratio for time-to-event OS prediction. Initially, clinical parameters were derived from the training cohort. These were integrated with radiomic features extracted from the primary gross tumor volume (GTV) outlined on the pre-treatment MRI scans. The risk-stratification model signatures were crafted within the training cohort and later assessed in the validation cohort.

To measure the accuracy of the model in stratifying patients into low and high-risk groups, we used a number of tools including the concordance index (C-Index) and the integrated area under the time-dependent ROC curve (iAUC) at 11 months introduced by our clinicians and model calibration.

### 2.3. Image Pre-Processing and Feature Extraction

The BraTS scans, originating from 19 different institutions, were acquired using diverse clinical protocols and scanners. Image pre-processing involved several steps for this dataset. First, MRI scans were converted from DICOM to NifTI format. Then, N4 bias correction was applied to scans as a temporary preparatory step for registration [25]. T1, T2 and FLAIR scans were registered to the T1ce sequence. Next, T1ce was registered to the SRI24 anatomical atlas [26], yielding co-registered, resampled volumes with uniform 1 × 1 × 1 mm^3^ isotropic voxel dimensions. A pre-trained deep learning model was used for brain tissue extraction from all scans, followed by intensity Z-scoring normalization. All steps were executed using the Cancer Imaging Phenomics Toolkit (CaPTk) [27]. For each MRI scan, the voxel resolution was fixed to 1 × 1 × 1 mm^3^ and the matrix size was fixed to 240 × 240 × 155.

For the image pre-processing of the STORM_GLIO dataset, we adopted techniques similar to those employed in curating the BraTS 2020 dataset. These included (1) skull stripping utilizing the HD-BET algorithm [28] and (2) applying rigid registration of all sequences to align with the T1ce modality, which is a previously validated workflow [29,30]. The MRI scans were uniformly resampled using B-splines to an isotropic voxel size of 1 × 1 × 1 mm^3^. The size of the resampled MRI images varied, as reported in the Supplementary Materials Table S1. The image pre-processing pipeline and settings were performed based on the guidelines established by the Image Biomarker Standardization Initiative (IBSI) [31].

Within the BraTS 2020 challenge, three distinct tumor regions were identified: enhancing tumor (ET), tumor core (TC; including both enhancing tumor and necrotic regions) and whole tumor (WT; comprising enhancing tumor, necrotic and edema). The STORM_GLIO dataset included manually delineated GTV segmentation, defined as the visible extent of malignant growth [32]. Based on previous validations [30,31], GTV and TC were treated as analogous regions for radiomic assessment. Using the four MRI scans associated to each patient, a total of 660 (4 × 165) imaging features were derived using the MATLAB version of Spaarc Pipeline for Automated Analysis and Radiomics Computing (SPAARC, https://www.spaarc-radiomics.io/, accessed on 1 July 2024) [33,34]. These imaging features are a large set of numerical indicators that describe various aspects of the characteristics of the tumor, such as its shape, texture and intensity patterns. All features, which were standardized following IBSI guidelines [31], were extracted using a 3D approach. The image pre-processing settings and collected radiomic features are summarized in Supplementary Figure S1.

### 2.4. Stability Analysis

The robustness of the radiomic feature against variations such as acquisition parameters and patient positioning were evaluated using image augmentation techniques such as those used by Zwanenburg et al. [35]. In this study, GTVs underwent rotations (−4°, −2°, 0°, 2°, 4°) and volume changes (−20%, −10%, 10%, 20%) in the training cohort. This generated a set of 20 variant images per patient for feature stability analysis. The intra-class correlation coefficient (ICC) with a 95% confidence interval (CI) was computed for each feature to assess consistency across such variations. When building the model, features with an ICC below 0.75 at the lower bound of the 95% CI were discarded. The same exclusion criteria were applied to the features extracted from the validation cohort.

### 2.5. Identifying a Clinical and Radiomic Signature

**Figure 2:**
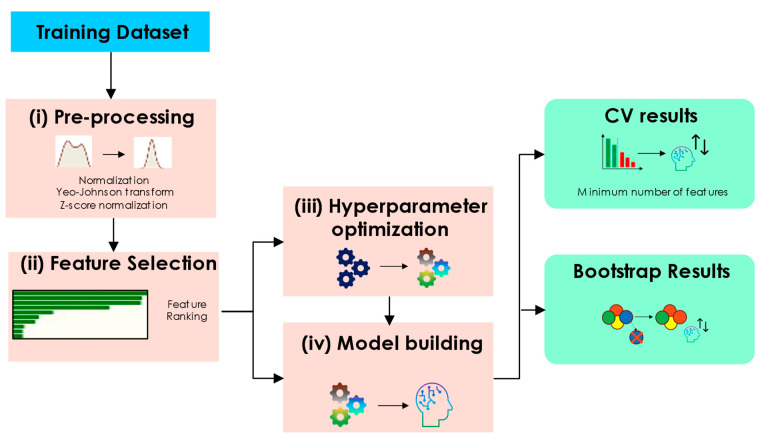
Overview of the framework used for feature selection and hyperparameter optimization.

For the time-to-event task, three feature selection methods were used, helping to avoid overfitting and enhance the model’s generalizability to new, unseen datasets. The fundamental steps of the workflow are shown in Figure 2: (i) feature pre-processing, (ii) feature selection (detailed workflow in Supplementary Materials Figure S2), (iii) hyper-parameter optimization for the ML algorithms, (iv) model building with internal validation. With the exception of (iv), all steps were employed with three-fold cross validation and 33 repetitions on the training data following the approach used by Kim et al., 2009 [36].

(i) Yeo-Johnson transformation was utilized to align the feature distributions with a normal distribution [37]. Then, features were z-score normalized. Both the transformation and the normalization were applied to the training dataset. The parameters derived from these processes were utilized to normalize the features in the validation dataset.

(ii) Following the approach used by Leger et al., 2017 [38], three feature selection methods were used: mutual information (MutInfo) [39], minimal redundancy maximum relevance (MRMR) [40] and regularized cox regression (Lasso) [41]. After feature selection, three prognostic models were used: regularized Cox regression (Cox-Lasso), gradient boosting survival (GBS) and random survival forests (RSF) [42]. These models are specifically designed for survival analysis, offering complementary approaches to analyzing the data and potentially enhancing the accuracy and robustness of the risk stratification.

(iii) To handle overfitting, hyperparameter-tuning was conducted through bootstrap sampling of the training datasets for each model.

(iv) To adhere to the radiomic guideline and meet the minimum feature number requirement of three features, including clinical information (age) [18], the two features collected and counted from each of the 99 cross-validation runs were ranked according to their frequency of occurrence.

The prognostic models built using three features were evaluated on 200 bootstraps of the entire training dataset to evaluate their stratification performance with the C-index.

The workflow was applied to build prognostic models on the training dataset. The developed prognostic models were tested on the validation dataset.

### 2.6. Statistical Analysis

The survival distributions of training and validation datasets were compared using the log-rank test. The χ^2^ test was employed to assess whether there were significant differences in the distribution of categorical variables within the clinical data between the training and validation cohorts. Continuous variables were assessed using the Mann–Whitney U test.

The risk scores derived from prognostic models were evaluated using Kaplan–Meier curve survival analysis, with the median risk score serving as the threshold (cut-off) to categorize patients into low and high-risk groups. The Kaplan–Meir curve was assessed by the log-rank test. The stratification performance of the prognostic models was evaluated by calculating the C-index. For calculating 95% CI, the C-index was evaluated using the 200 bootstraps on the training and the validation cohorts [43]. Furthermore, the integrated area under the time-dependent ROC curve (iAUC) was calculated [44]. While the conventional AUC assesses event status and predictor value for each patient at fixed points over time, iAUC measures the incremental change over time. We also calculated iAUC at eleven months for all models as determined by our clinicians. Statistical and survival analyses were performed with Python software version 3.9. A *p*-value < 0.05 was considered a statistically significant difference. The image preprocessing and statistical analysis workflow are shown in Figure 2. Permutation feature importance using the Sklearn library v1.3.2 was utilized to show feature importance (cf. Supplementary Materials, Table S2).

## 3. Results

Clinical characteristics of both the training and validation cohorts are shown in Table 1. The median value of OS was 11.9 months for the training cohort and 12.3 months for the validation cohort. The OS data between the two cohorts did not show a significant difference (*p* = 0.48, Table 1). Out of 660, 523 stable RFs remained after the robustness analysis. All RFs demonstrated a weak correlation with age, with correlation coefficients below 0.3 (Spearman < 0.3). In total, 227 RFs remained after excluding those with a high correlation (Spearman > 0.95). The robust RFs were used to conduct feature selection workflow via the three-fold cross-validation setting with 33 repetitions (99 runs). A pool of 37 RFs were identified using Lasso feature selection. The top two RFs were selected from the feature set by ranking occurrence frequency. For the top two RFs and age, 200 bootstrapping on the entire training cohort was applied to select the hyper parameters of each three-feature prognostic model. The selected hyperparameters settings can be found in Supplementary Materials Table S3.

**Figure 3:**
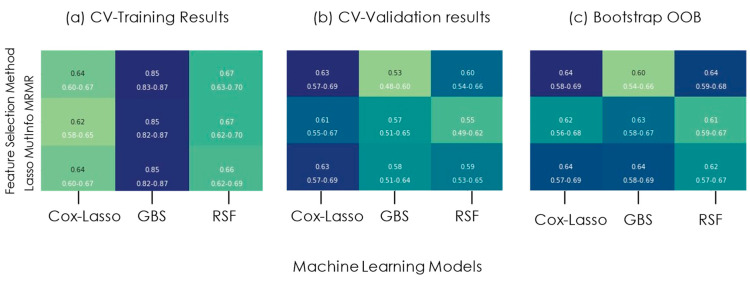
C-index of models based on each feature selection method and each corresponding machine learning algorithm for the prognosis of GBM. (**a**) CV-Training results. (**b**) CV-Validation results. (**c**) Bootstrap OOB.

The clinical–radiomic signature based on age and two RFs was utilized to build prognostic models on the training cohort. The top two RFs selected by the feature selection methods are shown in Table 2. The optimal performing RFs, which need the minimum number of MRI modalities, were achieved by the Lasso feature selection method. Additionally, the Cox–Lasso model, which is the most interpretable model among various machine learning models according to Luo et al. [45], exhibits a C-index of 0.64 as shown in Figure 3c.

In the training cohort, the radiomic model had optimum results using only two RFs: morph_av (morphological, occurrence: 31%) and dzm_zdnu_3D (texture, occurrence: 16%). The model had a C-index of 0.60 (95% CI: 0.54–0.66) and a Hazard Ratio (HR) of 2.72 (95% CI: 1.66–4.46). The two RFs were both derived from the FLAIR modality and exhibited weak correlation with each other (Spearman < 0.6). Morph_av (IBSI: 2PR5) is a shape-based feature providing surface-to-volume ratio. Dzm_zdnu_3D (IBSI: V294) is a texture feature quantifying the association between spatial location and grey level value by measuring the size of homogeneous zones (groups) within a specified distance. It captures the distribution of such zone counts across various distances. It is derived from the Grey Level Distance Zone Matrix (GLDZM).

In the validation dataset, the radiomic model had the best C-index (0.62, 95% CI: 54–71), and HR (2.97, 95% CI: 0.8–10.99) as reported in Table 3. In the training dataset, the clinical–radiomic model, using a clinical feature and RFs, had the best C-index (0.63, 95% CI: 0.56–0.74). As reported in Table 3, this model had a C-index (0.69, 95% CI: 0.62–0.75) in the validation dataset.

**Figure 4:**
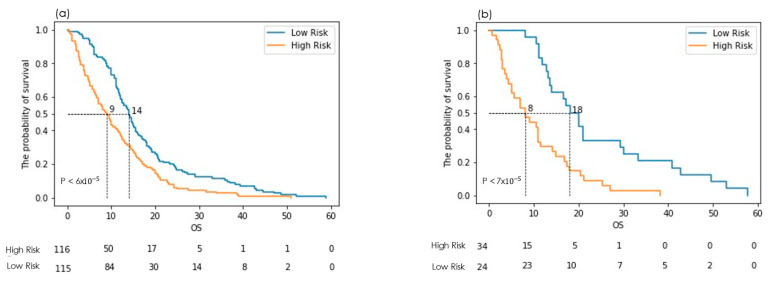
Kaplan–Meier plots showing differences between (**a**) training and (**b**) validation datasets stratified into low or high-risk groups by the Cox–Lasso model. The small *p*-values indicate a highly reliable differentiation between the risk groups.

The cut-off point for the Kaplan–Meier curve was 0.015 (c.f. Supplementary Materials Table S4). The log-rank *p*-value was 6 × 10^−5^ in the training dataset. For the same cut-off value, the log-rank *p*-value was 7 × 10^−5^ in the validation dataset (Figure 4a,b). Kaplan–Meier plots clearly demonstrate the model’s consistent ability to distinguish between high and low-risk groups across both training and validation datasets. The distinct separation between the survival curves, coupled with the highly significant *p*-values, underscores the model’s reliability and potential predictive power for diverse, unseen patient populations. This robust performance suggests the model could be a valuable tool for tailoring prognoses and developing personalized treatment strategies based on individual risk profiles.

The iAUC at 11 months of the prognostic model with only two RFs had 0.63 in the training dataset and 0.78 in the validation dataset. The iAUC at 11 months of the model with only the age variable achieved 0.62 in the training dataset and 0.67 in the validation dataset.

The iAUC at 11 months of the clinical–radiomic model incorporating age and two RFs was 0.69 in the training dataset and 0.81 in the validation dataset.

**Figure 5:**
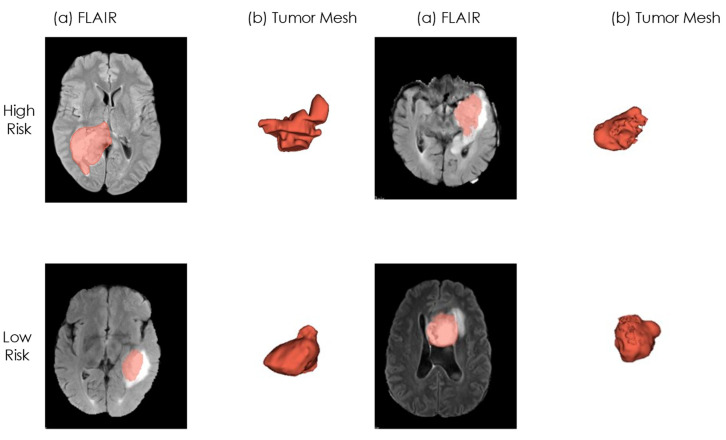
The visualization of risk groups (first row: high risk, second row: low risk) for median OS values of each group. For each case, a transverse slice from the FLAIR scan (**a**) is coupled with a 3D mesh of the tumor. (**a**) FLAIR (**b**) Tumor Mesh.

In Table 3, HR shows the most significant impact from the GLDZM-based feature with a value of 1.89. Age and morphology features demonstrate almost identical effects, with values of 1.60 and 1.58, respectively. Figure 5 shows a visual representation of both risk groups on example cases.

## 4. Discussion

In this study, we developed a clinical–radiomic prognostic model to stratify GBM patients into low and high-risk groups by using preoperative MRI. It is important to note that converting from the RTSTRUCT to mask can influence the radiomics analysis by using a different software platform [46]. Therefore, we consistently used a single software (Python) for generating masks in the STORM_GLIO dataset. Using robustness analysis of radiomic features, feature selection methods provided two RFs derived from only the FLAIR modality. The clinical–radiomic model was validated with a C-index of 0.69 with significant differences on the stratified risk groups.

In Table 4, we compare the findings of our study with those reported in previous investigations. We applied specific inclusion criteria: studies must use only radiomics features, focus on GBM (Grade 4) and work on time-to-event tasks (overall survival). Studies not meeting these criteria were excluded. The table reveals potential biases related to patient sample size, specifically limited patient cohorts and single-center studies, which may compromise the validity and reliability of the findings. For instance, while Hajianfar et al. [17] reported the highest C-index, their study had the smallest patient cohort. To mitigate this, we aimed to maximize our patient cohort from multiple centers. However, potential bias risks remain in our study as well. It can be noted that Cepeda et al. [15] reported a model built on multiple MRI modalities and 10 RFs (C-index = 0.61, and iAUC = 0.77). A similar result (AUC = 0.75) with 57 RFs was obtained by Tixier et al. [14]. Verma et al. achieved a comparable performance (AUC = 0.78) with over 300 features and multiple MRI modalities [16]. Additionally, Hajianfar et al. reported a C-index of 0.77 [17] for a model based on the smallest patient cohort and using convolutional filters which were not IBSI standardized at the time of publication [47]. Our study demonstrated a comparable C-index and the best iAUC at 11 months. This was accomplished by utilizing the largest patient cohort, employing the smallest number of RFs, deriving RFs from only the MRI FLAIR modality and utilizing only one ROI (GTV).

Exploring several combinations of feature selection and machine learning algorithms, we showed that the use of age, alongside one modality-independent morphology feature (morph_av) and one GLDZM feature (dzm_zdnu_3D) from MRI FLAIR modality, yielded the most favorable outcomes for generalizability on the validation set. This enables the model to perform well in diverse healthcare settings, ranging from small local clinics to large research hospitals. It provides dependable predictions and valuable insights using data from various sources, highlighting its adaptability and reliability across different medical environments. As can be seen in Figure 5b for the interpretation of RFs, the high-risk patients can be characterized by very irregular boundaries and a non-smooth, irregular shape. This means having a higher surface area to volume ratio (‘morph_av’). Additionally, Zone Distance Non-Uniformity derived (ZDNU) from GLDZM measures the variability of zone sizes and distances in a 3D image (dzm_zdnu_3D). In addition, patients in the high-risk category have a higher ZDNU value which is attributed to a more heterogeneous textural pattern (c.f. Figure 5a). This highlights that even regions appearing homogeneous can display considerable variations in zone size at different distances. The combination of RFs with age resulted in enhanced outcomes compared to using only clinical information for GBM. This is a finding also reported by Cepeda et al. [15]. Integrating clinical (age) and radiomic data can improve model performance, but it risks obscuring the significance of clinical factors. Without well-structured feature selection and model building processes, models may overfit the training data and perform poorly on new datasets, highlighting the need for a careful balance between clinical and radiomic features. In the medical domain, data sparsity/scarcity and imbalance pose significant challenges due to the rarity of certain diseases, limited cohort sizes and missing clinical information, making it difficult to collect comprehensive datasets [48]. In our research, we encountered similar challenges in collecting comprehensive data, including various MRI sequences (T1, T1ce, T2 and FLAIR) and multiple clinical parameters such as age, genetic information, survival metrics and Karnofsky performance status. Acknowledging these clinical constraints, we aimed to maximize the patient cohort by collecting a minimal number of clinical data, extracting radiomics features from a minimal set of MR sequences. This approach was targeted to achieve an optimal balance between data availability and model performance. Additionally, the potential bias associated with the retrospective dataset was reduced by collecting a multi-center patient cohort with the largest achievable sample size.

The performance of our model could be increased by using additional labels besides GTV, such as the multiple regions of feature extraction used in previous studies, as shown in Table 4. Additionally, deep learning (DL)-based features could be employed to enhance performance in survival analysis. However, this study excluded deep radiomic features due to their low reproducibility and interpretability which are important limitations for clinical applications [49].

Previous studies did not prioritize minimum requirements, such as using a singular ROI or maximizing interpretability by minimizing the number of radiomic features. On the other hand, our work is in line with recommendations by van Timmeren et al. [18] suggesting that the number during building a radiomic model should be limited to the range [3,10]. To mitigate overfitting, a workflow was designed including hyperparameter optimization and resampling of the data. We reported the results of the prognostic model on the independent validation dataset with this workflow.

## 5. Conclusion

In this study, we developed and validated a clinical–radiomic model for stratification of GBM patients according to OS. To the best of our knowledge, this is the first study to utilize MRI-based RFs following the IBSI guidelines while prioritizing clinical challenges, interpretability issue and robustness analysis for GBM. This approach has resulted in superior performance compared to previous studies as reported by Tabassum et al., 2023 [11]. Our model utilized two independent RFs from FLAIR MRI modality and age. Due to recent standardization of convolutional filters by the IBSI consensual guidelines [47], we aim to use convolutional filters for RFs in the future. Furthermore, work is in progress at our institution to explore the use of DL features to enhance performance, subject to the verification of their interpretability. This could involve exploring the application of multimodal foundation models, integrating additional clinical factors such as age, sex, Karnofsky performance status or incorporating multi-modality imaging data (PET, CT etc.). Other potential areas for performance improvement include incorporating diffusional or functional MRI sequences and collecting more comprehensive clinical information, including omics data such as genomics, transcriptomics, and metabolomics, to develop more accurate and reliable models, which could be facilitated by larger patient cohorts.

## Figures and Tables

**Table 1:** Characteristics of clinical variables for training and validation datasets.

Variable	Training Dataset Median (Range)	Validation Dataset Median (Range)	Statistical Cohort Comparison
**Age (years)**	61.1 [18.98–86.27]	63.4 [31.0–86.65]	U: 0.65, *p*-value: 0.74
**OS (months)**	11.9 [0.17–58.9]	12.3 [0.7666–57.7]	U: 0.63, *p*-value: 0.48
**OS < 11-month (%)**	43.7% (101/231)	39.7% (23/58)	χ^2^: 0.19 *p*-value: 0.66

**Table 2:** The selected feature names are shown for each feature selection method. Each feature is displayed with its dependent modality in parentheses, except for “morph_av”, which is a modality-independent feature.

Feature Selection Method		
Lasso	MutInfo	MRMR
morph_av dzm_zdnu_3D (FLAIR)	szm_glnu_3D (T1ce) stat_p10 (T2)	dzm_zdnu_3D (FLAIR) szm_glnu_3D (T1ce)

**Table 3:** Univariate and Multivariate Cox regression analysis.

Univariate Cox Regression Analysis							
Dataset	Model	Variable	HR [95% CI]	*p*-Value	C-Index	iAUC	11m-iAUC
Training	Clinical model	Age	1.32 [1.15–1.50]	0.010	0.59 [0.53–0.64]	0.67	0.62
	Radiomic model	RFs Risk Score	2.72 [1.66–4.46]	0.007	0.60 [0.54–0.66]	0.67	0.63
Validation	Clinical Model	Age	1.63 [1.23–2.16]	0.006	0.63 [0.56–0.68]	0.66	0.67
	Radiomic model	RFs Risk Score	2.97 [0.8–10.99]	0.290	0.62 [0.54–0.71]	0.79	0.78
**Multivariate Cox Regression Analysis**							
**Dataset**	**Model**	**Variable**	**HR [95% CI]**	***p*-Value**	**C-Index**	**iAUC**	**11m-iAUC**
Training	Clinical–radiomic Model	Age	1.30 [1.14–1.49]	6 × 10^−5^	0.63 [0.56–0.74]	0.68	0.69
		morph_av	1.02 [0.87–1.20]				
		dzm_zdnu_3D	1.36 [1.13–1.62]				
Validation	Clinical-radiomic Model	Age	1.60 [1.21–2.13]	7 × 10^−5^	0.69 [0.62–0.75]	0.78	0.81
		morph_av	1.58 [1.08–2.29]				
		dzm_zdnu_3D	1.89 [1.19–3.01]				

**Table 4:** The comparison of recent similar studies with our study.

References	No. of Patients	MRI Sequence	Region of Feature Extraction	Extracted Feature Number	Selected Feature Number	Feature Number Guideline (3–10)	ML Model	Validation Method	IBSI Guideline	Performance Metrics
Tixier et al. [14]	234	T1	Gd-ET, NEC, NET, TC	88	57	No	Lasso	Five-fold CV	Yes	AUC: 0.75
Cepeda et al. [15]	203	T1ce, T1, T2, FLAIR	Tumor, Peritumoral	15,720	10	Yes	Random Forest Survival	Five-fold CV	Partially (Convolutional Filters)	iAUC: 0.77 C-index 0.61
Verma et al. [16]	150	T1ce, T2, FLAIR	ET, NCR	3792	316	No	-	Five-fold CV	Partially (Convolutional Filters)	AUC: 0.78
Hajianfar et al. [17]	119	FLAIR, T1ce	ET, TC, NEC, ED	4471	-	No	Cox Boost	Three-fold CV Bootstrap	Partially (Convolutional Filters)	C-index: 0.77
Our Study	289	FLAIR	GTV (TC)	689	2 (without Age)	Yes	Cox-Lasso	Three-fold CV 33 repetitions Bootstrap	Yes	C-index: 0.69 iAUC: 0.81

## Data Availability

The BraTS data used in this study are openly available and can be accessed from the following source: [21].

## Supplementary Materials

The following supporting information can be downloaded at: https://www.mdpi.com/article/10.3390/cancers16193351/s1, Figure S1: Settings in IBSI compliant terminology for radiomics analysis carried out with the SPAARC code. Figure S2: Feature Selection Workflow: Correlation analysis using Spearman and Pearson methods, feature selection through Lasso Cox, MRMR, and Mutual Information, ranking features over multiple iterations, feeding the model with the selected features. Table S1: Selection of relevant MRI acquisition parameters for the scans included the STORM_GLIO dataset. Table S2: Permutation feature importance: Permutation feature importance was conducted (Sklearn v1.3.2 in Python) test for 200 repetitions. Table S3: The selected hyperparameters settings from 200 bootstrapped iterations of the training dataset. Table S4: Feature weights and cut-off value. The weight of each feature and the cut-off value for risk-stratification into low and high-risk groups.
