# Supplementary material for: Reproducible and Interpretable Machine Learning-Based Radiomic Analysis for Overall Survival Prediction in Glioblastoma Multiforme
Source: Cancers (Basel). 2024 Sep 30;16(19):3351. doi: 10.3390/cancers16193351 (PMC11476262; doi:10.3390/cancers16193351)
Supplement: Supplementary file 1 [file cancers-16-03351-s001.zip › cancers-3215423-supplementary.pdf]

Supplementary Materials:

Reproducible and Interpretable Machine Learning-Based Radiomics Analysis for Predicting Overall Survival in Glioblastoma Multiforme

Contents

A. MRI Acquisition Parameters collected from DICOM for STORM_GLIO 3

B. Feature Selection Workflow 4

C. Feature Importance 4

D. Hyperparameter Optimization 5

E. Feature weights and cut-off value 5

References ....

A. MRI Acquisition Parameters collected from DICOM for STORM_GLIO

Table S1 Selection of relevant MRI acquisition parameters for the scans included the STORM_GLIO dataset.

	**T1**	**T1ce**	**T2**	**FLAIR**
Thickness (mm)	4.77 +-0.47	4.76 +-0.47	4.74 +-0.56	4.81 +-0.39
Repetition time (ms)	489 +-96	494 +-98	5627 +-1856	8084 +1832
Echo Time (ms)	11 +-2	11 +-2	97 +-8	112 +-27
Inversion Time (ms)	0 +-0	0 +-0	0 +-0	2217 +-259
Field Strength (T)	1.54 +0.24	1.5 +-0	1.54 +0.24	1.54 +0.24
Rows	426 +-145	424 +-146	546 +-185	475 +-219
Columns	417 +-147	415 +-148	527 +-198	458 +-232
Pixel spacing (mm)	0.62 +-0.19	0.62 +-0.19	0.48 +-0.14	0.59 +-0.21
Slice Spacing (mm)	5.99 +-0.73	5.98 +-0.74	6.27 +-0.96	6.34 +-0.72
SAR	1.09 +-0.77	1.07+- 0.76	0.91 +-0.53	0.69 +-0.67

```
{
  "interpolation": {
    "new_voxel_spacing": [1, 1, 1],
    "method": "spline",
    "rounding_after_interp": true
  },
  "feature_families": ["morph", "stats", "ih", "ivh", "glcm", "glrlm",
"glszm", "ngldm", "ngtdm", "gldzm"],
  "re_segmentation_range": {"min": "", "max": ""},
  "re_segmentation_outlier_filtering": {"apply": false, "sigma": 3},
  "bin_method": "FBN",
  "bin_value": 64,
  "analysis_type": "3D",
  "texture_parameters": {
    "glcm": {"aggregation": "merged", "distance": 1},
```

```
    "glrlm": {"aggregation": "merged", "distance": 1},
    "ngtdm": {"distance": 1},
    "ngldm": {"distance": 1, "alpha": 0}
  }
}
```

Figure S1 Settings in IBSI compliant terminology for radiomics analysis carried out with the SPAARC code.

B. Feature Selection Workflow

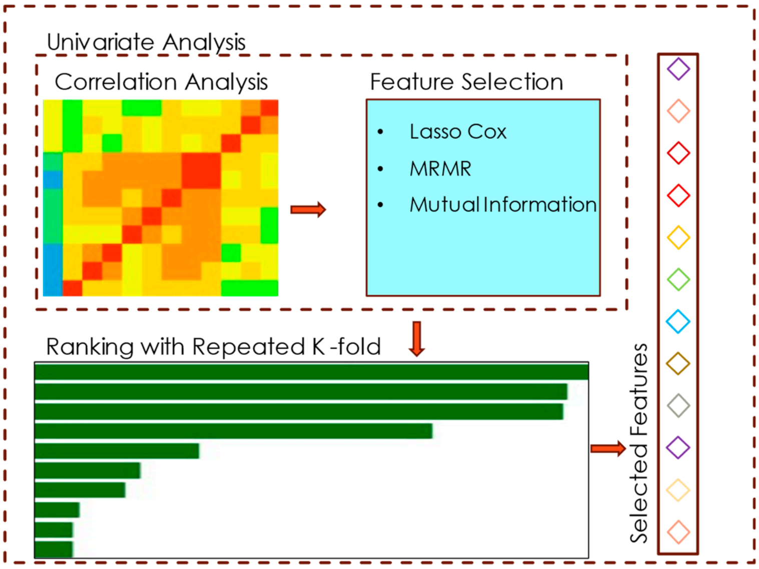

Figure S2 Feature Selection Workflow: Correlation analysis using Spearman and Pearson methods, feature selection through Lasso Cox, MRMR, and Mutual Information, ranking features over multiple iterations, feeding the model with the selected features.

C. Feature Importance

Table S2 Permutation feature importance: Permutation feature importance was conducted (Sklearn v1.3.2 in Python) test for 200 repetitions

Feature Selection Method	Feature Names	Machine Learning Model
		Cox-Lasso
Lasso	morph_av	0.001
	dzm_zdnu_3D (FLAIR)	0.07
	Age	0.06
MRMR	dzm_zdnu_3D (FLAIR)	0.03
	szm_glnu_3D (T1ce)	0.02
	Age	0.06
Mutational Information	stat_mean (T1)	0.01
	cm_joint_entr_3D_comb (FLAIR)	0.02
	Age	0.09

D. Hyperparameter Optimization

Table 3 displays the selected hyperparameters derived from 200 bootstrapped iterations of the training dataset.

Table S3 The selected hyperparameters settings from 200 bootstrapped iterations of the training dataset.

**Model**	**Feature Selection**	**Hyperparameters (selected [min,max])**
Cox-Lasso	Mutual Information	alpha = 2 [1,5]
Cox-Lasso	MRM	alpha = 2 [1,5]
Cox-Lasso	Lasso	alpha = 2 [1,5]
GBS	Mutual Information	n_estimators = 2 [1,5]<br>max_depth = 2 [1,5]<br>min_samples_split =2 [1,5]
GBS	MRM	n_estimators = 2 [1,5]<br>max_depth = 2 [1,5]<br>min_samples_split = 2 [1,5]
GBS	Lasso	n_estimators =2 [1,5]<br>max_depth = 2 [1,5]<br>min_samples_split = 2 [1,5]
RSF	Mutual Information	n_estimators = 2 [1,5]<br>max_depth = 2 [1,5]<br>min_samples_split = 2 [1,5]
RSF	MRM	n_estimators = 2 [1,5]<br>max_depth = 2 [1,5]<br>min_samples_split = 2 [1,5]
RSF	Lasso	n_estimators = 2 [1,5]<br>max_depth = 2 [1,5]<br>min_samples_split = 2 [1,5]

E. Feature weights and cut-off value

Table S4 Feature weights and cut-off value. The weight of each feature and the cut-off value for risk-stratification into low and high-risk groups.

		**Weights**
**Feature Name**	morph_av	-0.107019
	dzm_zdnu_3D (FLAIR)	0.208010
	Age	0.340629
**Cut-off Value**	Median value of risk scores	0.015
